# 

**Published:** 1819-10

**Authors:** 


### LECTURES ANNOUNCED TO COMMENCE IN OCTOBER.

Conquest (Dr.) on Midwifery.
Curtis (Mr.) on the Anatomy, Physiology, and Pathology, of the Ear.
Davies (Dr. J. B.) on the Diseases and Medical Management of Children and young Persons.
——— (Dr.) on the Practice of Midwifery.
*Glasgow College.*—Mr. Burns, on Surgery; Dr. Freer, on the Practice of Medicine, and on the Institutions of Medicine; Dr. Graham, on Botany, in May next; Dr. Jeffery, on Anatomy; Dr. Miller, on Materia Medica and Pharmacy; Mr. Towers, on Midwifery; Dr. Thomson, on Chemistry and Chemical Pharmacy.
Ramsbotham (Dr.) on the Practice of Midwifery.
Uwins (Dr.) Introductory Lecture on the Practice of Medicine.

### MONTHLY CATALOGUE OF MEDICAL BOOKS.

Some Remarks on an Introductory Lecture delivered before the Royal College of Surgeons, in which an Attack was made on a Teacher of Anatomy in one of the regular Schools of London. By a Member of the College, late a Pupil of the School of Great Windmill-street. 8vo. 2s.

A Treatise on Stricture of the Urethra, containing an Account of improved Methods of Treatment; with an Appendix, noticing the Applications of a new Instrument to the enlarged Prostate Gland, Gleet, Fistula, and other Diseases of the Urethra, Œsophagus, and Rectum. By James Arnott, Surgeon. 8vo. boards, 7s.

A Letter to the Right Hon. Lord Viscount Palmerston, Secretary at War, &c. on the subject of the Ophthalmic Institution, for the Cure of Chelsea Pensioners. By John Vetch, M.D. F.R.S.E. Physician to the Forces, &c. &c. The second Edition, with an additional Appendix, containing the Reports of the Army Medical Board, and of the Medical Officers of Chelsea Hospital. 2s. 6d.

Catalogue of Mr. Charles Bell's Museum of Natural and Morbid Anatomy, in Great Windmill-street. 4to. 2s.

Clinical and Pathological Reports. By Samuel Black, M.D. 8vo. 10s. 6d.

Burgess and Hill's Improved Catalogue of Books in Anatomy, Medicine, &c.

### *TO SUBSCRIBERS.*

*The unequivocal testimony we have experienced, that the project of making the periodical History of Medicine form a Supplement to the common series of the Work is generally favoured by our readers, by the numerous expressions of approbation; and the want of a single instance of objection to that measure, has determined us to fulfil our proposal; but it is necessary to state, those who desire to obtain the Supplement, should give directions to that effect to their respective Booksellers, and some time previous to the period of its publication; as it will not be generally distributed with the Journal, unless required.*

